# Consumers’ knowledge and attitudes about food additives in the UAE

**DOI:** 10.1371/journal.pone.0282495

**Published:** 2023-03-06

**Authors:** Tareq M. Osaili, Reyad S. Obaid, Sanaa A. I. Alkayyali, Hind Ayman, Sara M. Bunni, Shaema B. Alkhaled, Fayeza Hasan, Maysm N. Mohamad, Leila Cheikh Ismail

**Affiliations:** 1 Department of Clinical Nutrition and Dietetics, College of Health Sciences, The University of Sharjah, Sharjah, United Arab Emirates; 2 Sharjah Institute for Medical Research, University of Sharjah, Sharjah, United Arab Emirates; 3 Department of Nutrition and Food Technology, Faculty of Agriculture, Jordan University of Science and Technology, Irbid, Jordan; 4 Department of Food Science, College of Agriculture & Veterinary Medicine, United Arab Emirates University (UAEU), United Arab Emirates; 5 Department of Nutrition and Health, College of Medicine and Health Sciences, United Arab Emirates University, Al Ain, United Arab Emirates; 6 Nuffield Department of Women’s & Reproductive Health, University of Oxford, Oxford, United Kingdom; University of Ilorin, NIGERIA

## Abstract

The use of food additives (FAs) in food manufacturing is a well-accepted practice worldwide. Inadequate knowledge concerning their safety may cause negative attitude surrounding their use. This would potentially impact the purchase of foods that the consumer perceives as containing FAs. This study aimed to assess knowledge and attitudes of consumers towards the use and safety of FAs in the UAE. A cross-sectional study was conducted using an online survey distributed via social media platforms (n = 1037). Less than one-third of the participants (26.7%) in this study stated that they knew what FAs are. About half the respondents believed that organic products did not contain FAs. The proportion of respondents who reported that the purpose of adding FAs is to extend shelf life, better the taste and aroma of food, enhance nutritional value, improve consistency and texture, and boost appearance and color was 92.1%, 75.0%, 23.5%, 56.6%, and 69.4%, respectively. Around 61% believed that all FAs were harmful to human health. The level of FA knowledge increased with age and education level. About 60% of the respondents reported that food labels did not provide sufficient information about FAs. The most preferred platforms for consumers to receive information about FAs were social media (41.1%), followed by brochures (24.6%). Overall, the UAE population had inadequate knowledge and a hesitant attitude concerning FAs. The municipalities and food industry should play an active role in educating the public to prevent and reduce any possible adverse attitudes towards processed food products.

## Introduction

As per the Food and Drug Administration (FDA), a food additive (FA) is any substance that is a component of the food and affects its characteristics [1]. Common food additives (FAs) like salt, oil, sugar, citrus juice, etc., have been used since time immemorial for food preservation purposes. In the past, additives were mostly in the form of naturally occurring substances/compounds. However, with the advancement of technology, synthetic FAs have found common ground in the food market. The ever-increasing demand for food that looks fresh/ appealing and has an extended shelf-life or improved safety has fueled their growth [2]. The FDA inventory lists the total number of FAs to be 3000 [1]. FAs which are nutrients such as vitamins and minerals are also added to foods for health reasons [3, 4]. An acceptable daily intake for FAs has been assigned jointly by the Food and Agriculture Organization (FAO) and the World Health Organization (WHO) [5]. The United Arab Emirates (UAE) along with other gulf cooperation council (GCC) countries follow the regulations of the GCC Standardization Organization (GSO). GSO Ministerial Committee has developed its own set of standards concerning ’Additives permitted for use in food stuffs’ (GSO 2500:2021). These standers assure that foods displayed in the market in the UAE are safe and suitable (i.e. properly labeled) under the mandate of Food Law No (02) of 2008.

Despite the boom FAs have given to the industry, several concerns regarding the long and short-term risks/ impacts of consuming FAs have been raised in the last couple of years. Their usage has been plagued with several health concerns ranging from allergy to cancer [6, 7]. Few studies which assess consumers’ knowledge and safety perceptions regarding FAs have been conducted previously [8, 9]. Inadequate knowledge could be associated with negative attitude concerning FAs [10]. This would potentially impact the purchase of foods that the consumer perceives as loaded with FAs [11]. A few examples include canned, frozen, or fermented foods. The climatic conditions of the UAE are not well suited for agriculture. Therefore, the country depends on imports to meet its population demand. An unfavorable attitude towards FA usage would result in increased demand for fresh, locally produced foods besides affecting the import economy. Therefore, it is important to analyze the knowledge, attitude, and practice of UAE consumers about FAs. It is also important to determine the needs and concerns of the population regarding information on FAs [12].

Previous research suggests that consumers are concerned about the potential health implications of food additive use and consumption [13, 14]. Moreover, a study investigating the consumer concerns about halal food additives and halal products in the UAE showed that 86.5% of consumers had a “great concern” that foods provided in the market may not be halal [15]. However, to the best of our knowledge, no other studies have assessed the knowledge, attitude, and practice towards the general use of FA in the UAE. Thereby, the objective of the present study was to assess FAs knowledge (with respect to FAs functions) besides their perceived trust and safety amongst consumers in the UAE. The study also investigated consumer need for further information requirements about FAs.

## Methods

### Survey questionnaire

A questionnaire was developed to inquire about consumers’ knowledge and safety perceptions regarding FAs. The questions/scales were adopted from previously validated surveys in published literature [8, 9, 13, 16, 17]. It consisted of 37 questions, categorized into 6 sub-sections. The original questionnaire was in English. For the ease of native Arabic speakers, the questionnaire was also translated to Arabic. Care was taken to include neutral words and avoid any leading sentences or answer choices.

The content of the questionnaire was validated by a panel of experts in the field of food safety who were fluent in both the languages. The experts were asked to note the time it took to complete the questionnaire and to rate the questions on a Likert scale of 1 to 10 for clarity, language, simplicity, ambiguity, the vocabulary used, and whether the question met the objectives of the study. Minor recommendations were made by the experts and any question which scored less than 70% in any of the studied aspects was either amended or removed. The total questionnaire response time was around 15–20 min. Besides this, a pilot study consisting of 30 participants was performed to ensure the reliability of the questionnaire. Data from the pilot test was not included in the results. A Cronbach’s Alpha of 0.8 was recorded and the questions were considered reliable.

The first section of the questionnaire included the socio-demographic information of the participants (6 questions). The second section was about the respondents’ information source concerning FAs and the level of trust they had on food labels (2 questions), while the third section pertained to the knowledge of participants regarding FAs (8 questions). The fourth section tackled the attitudes of consumers towards FAs (12 questions). The fifth section assessed the consumer behavior with FAs (2 questions), while the last section was about the respondents’ needs in terms of FAs labelling (6 questions). The full questionnaire is provided as a supporting file (S1 File).

## Study design and participants

A cross-sectional study design was used to collect data from people living in various emirates of the UAE. The data were collected over three months ranging from February to April 2021. The questionnaire was developed on Google forms. The questionnaire was disseminated via various social media platforms (Facebook™, WhatsApp™, and Instagram™) to ensure maximum coverage. Respondents were asked to further share the link with their acquaintances living in any of the seven emirates of the UAE (Abu Dhabi (capital), Dubai, Sharjah, Ras al Khaimah, Umm al Quwain, Ajman and Fujairah) to maximize responses (snowball sampling technique) [18].

A total of 1,037 participants completed the questionnaire. The minimum required sample number was calculated to be 385 (based on the sample size calculation for prevalence studies) [19]. A response distribution of 50%, confidence level of 95%, margin of error of 5% and total population of 9.89 million was considered. The current sample exceeded the minimum required number. The inclusion criteria were participants aged 18 years or older and residing in the UAE. The participants were chosen regardless of their gender, marital status, emirate within the country or ethnic disposition.

### Data analysis

The Statistical Package for Social Sciences for Windows (SPSS Inc., version 28.0 Chicago, IL, 2021) was used to analyze data collected from the survey. Descriptive analysis was conducted to calculate frequency, percentage, mean, and standard deviation for the data where applicable.

Scores were calculated by the summation of correct answers. Each correct answer in a scored question was given a single point. The maximum possible score was 60. The participants that scored 50% or higher were categorized as having ‘good knowledge and attitude’, while those who scored below 50% were categorized as having ‘poor knowledge and attitude’. The independent sample t-test and one-way ANOVA tests were carried out to examine differences in means of the knowledge and attitude scores by sociodemographic variables. Only variables with a *p*-value <0.05 were included in the final multivariate logistic regression tests. Statistical significance of *p* <0.05 was used for all tests.

### Ethical statement

This study was conducted according to the guidelines laid down in the Declaration of Helsinki and all procedures involving human subjects were approved by Research Ethics Committee at the University of Sharjah (REC-21-03-03-05-S). An electronic informed consent was obtained from all participants. A consent form addressing the aims of the study was included on the first page of the questionnaire. Only those who consented to participate in the study were able to view and answer the questions. The consent form clearly stated that the response was voluntary, and the respondents had the right to withdraw from the questionnaire at any given time. The survey did not require the respondents to identify themselves and all responses were treated with high confidentiality. The respondents were given the information of the authors conducting the study for any further concerns/questions. Only consenting participants were directed to the next part of the questionnaire.

## Results and discussion

### Sociodemographic characteristics

The majority of the respondents in this study were female (78.9%) and belonged to the age category of 18–24 years (30.8%) followed by 26.4% from 25–34 years and 24.1% from 35–44 years, respectively (Table 1). The major constituent ethnic group in this study was that of Arabs (92.7%) with 90.9% of the respondents having a bachelor’s degree or higher. A study conducted previously indicated a higher level of education was synonymous with superior knowledge concerning FAs [8]. About 40% of the respondents in this study reported being single. Around half of the sample did not report having children (46.1%).

**Table 1:** Socio demographic characteristics of the respondents.

Demographic variable		Frequency	Percentage (%)
**Gender**	Female	819	78.9
	Male	219	21.1
**Age**	18–24	320	30.8
	25–34	274	26.4
	35–44	250	24.1
	45–60	183	17.6
	>60	11	1.1
**Nationality**	Arab expat	962	92.7
	Non-Arab expat	76	7.3
**Education**	High school or less	95	9.2
	Bachelor’s	777	74.9
	MSc or PhD	166	16
**Marital Status**	Single	411	39.6
	Married	598	57.6
	Widow	12	1.2
	Divorced	17	1.6
**No. of children**	0	479	46.1
	1	90	8.7
	2	147	14.2
	3	144	13.9
	4	107	10.3
	5 or more	71	6.8

**Fig 1:**
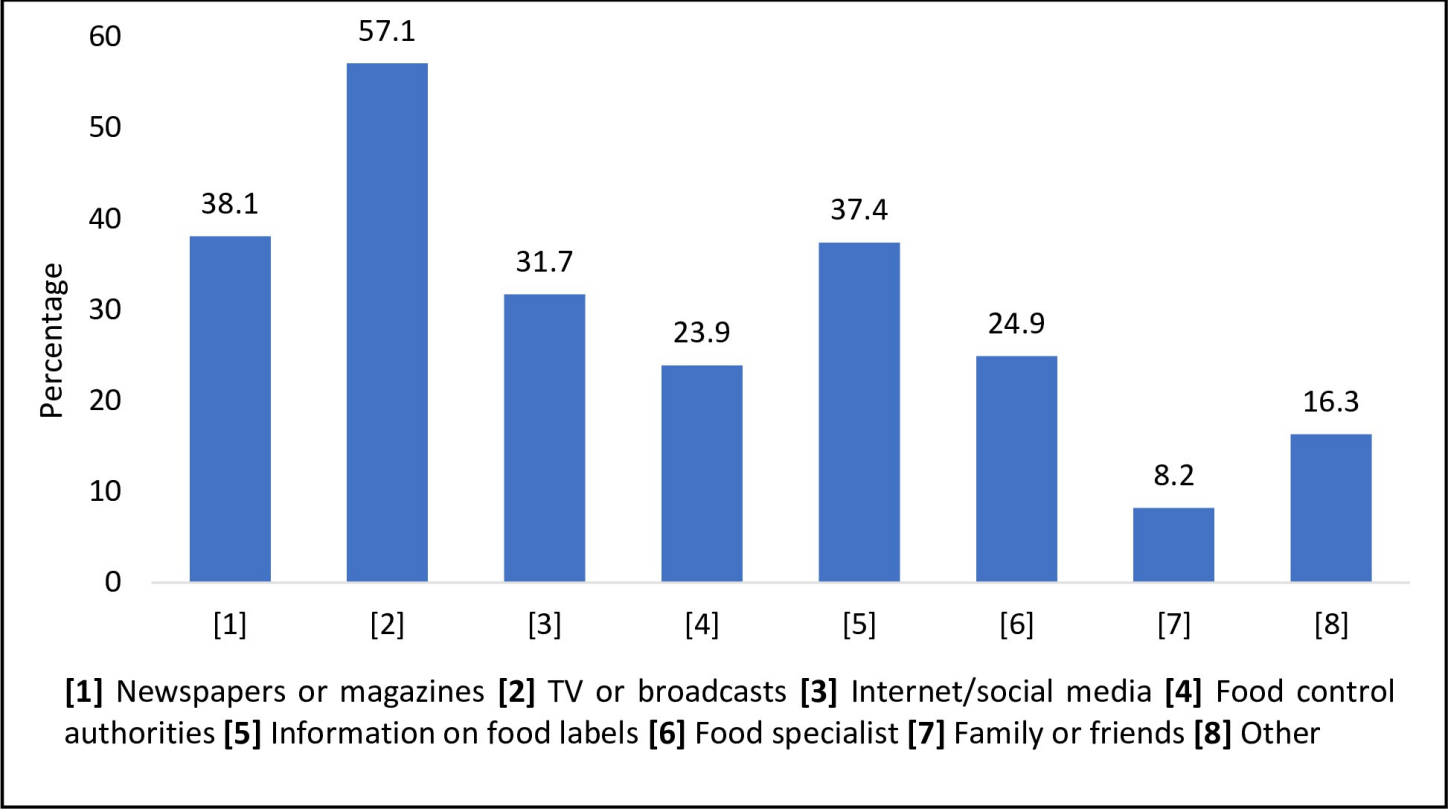
The different sources of respondent knowledge about food additives (n = 1038).

With respect to the sources of information about FAs, 14.4%, 18.8%, 69.3%, 19.8%, 63.4%, 36.4%, 28.7% of the respondents stated that newspapers/ magazines, TV/ broadcasts, internet/ social media, food control authorities, information on food labels, food specialists, and family and friends were their sources to learn about FAs (Fig 1). Similar statistics about the source of information about FAs were observed in a previous study [20]. The respondents in that study used radio and TV programs (56%), the internet (42%), and other sources (4%) to gain knowledge about FAs. Similarly, mass media was observed to be the most effective way of transmitting information concerning FAs (59.5%) [9]. Consumers in Europe were observed to trust the information given in science books [21]. It was observed in this study, that most participants (46.2%) adopted a neutral stance when it came to trusting food labels and only 34.9% of the respondents trusted them to a high degree. Media coverage about FA, the place of manufacture of the food product, knowledge about the local regulatory food inspection were all observed to influence consumer trust on food labels [22].

### Knowledge about types, function, and safety of FAs

**Fig 2:**
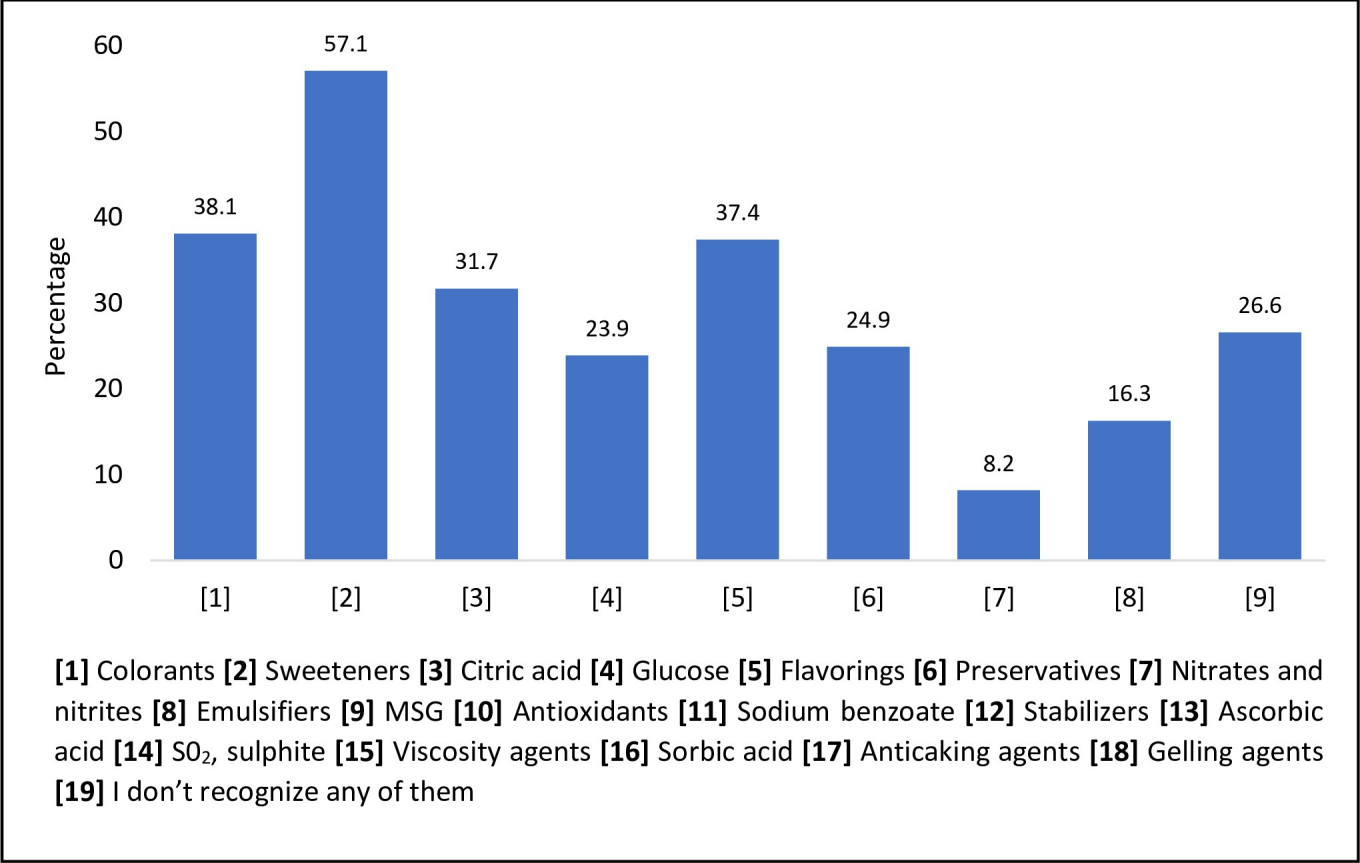
The percentage of respondents who recognize the different food additives (n = 1038).

Less than one-third of the participants (26.7%) in this study stated that they knew what FAs are. In terms of the ability of the respondents to recognize FAs, the percentage of respondents who believed they recognized colorants, sweeteners, citric acid, glucose, flavorings, and preservatives in food was between 54.3% to 84% (Fig 2). Similarly, 14.6% to 39.0% of the respondents reported recognizing nitrates and nitrites, emulsifiers, MSG, antioxidants, sodium benzoate, stabilizers, ascorbic acid, sulphites, viscosity agents, sorbic acid, anticaking agents, and gelling agents (Fig 2). In terms of the knowledge regarding the uses of FAs, less than a quarter (33.6%) of the respondents answered correctly that monosodium glutamate (MSG) is a flavor enhancer. This is lower than a study conducted in Mauritius, where 44.4% of the respondents knew that MSG is a flavor enhancer [23]. On the other hand, a good percentage (80.5%) knew that gelatin is a gelling agent. In contrast to a previous study, 40% and 30% of the respondents did not recognize FAs such as MSG and sodium salt of nitrite [20]. In this study, only 25.7% were aware that nitrates are coloring agents used in foods. Consumers who are educated about FAs have been observed to purchase less processed foods [24].

**Fig 3:**
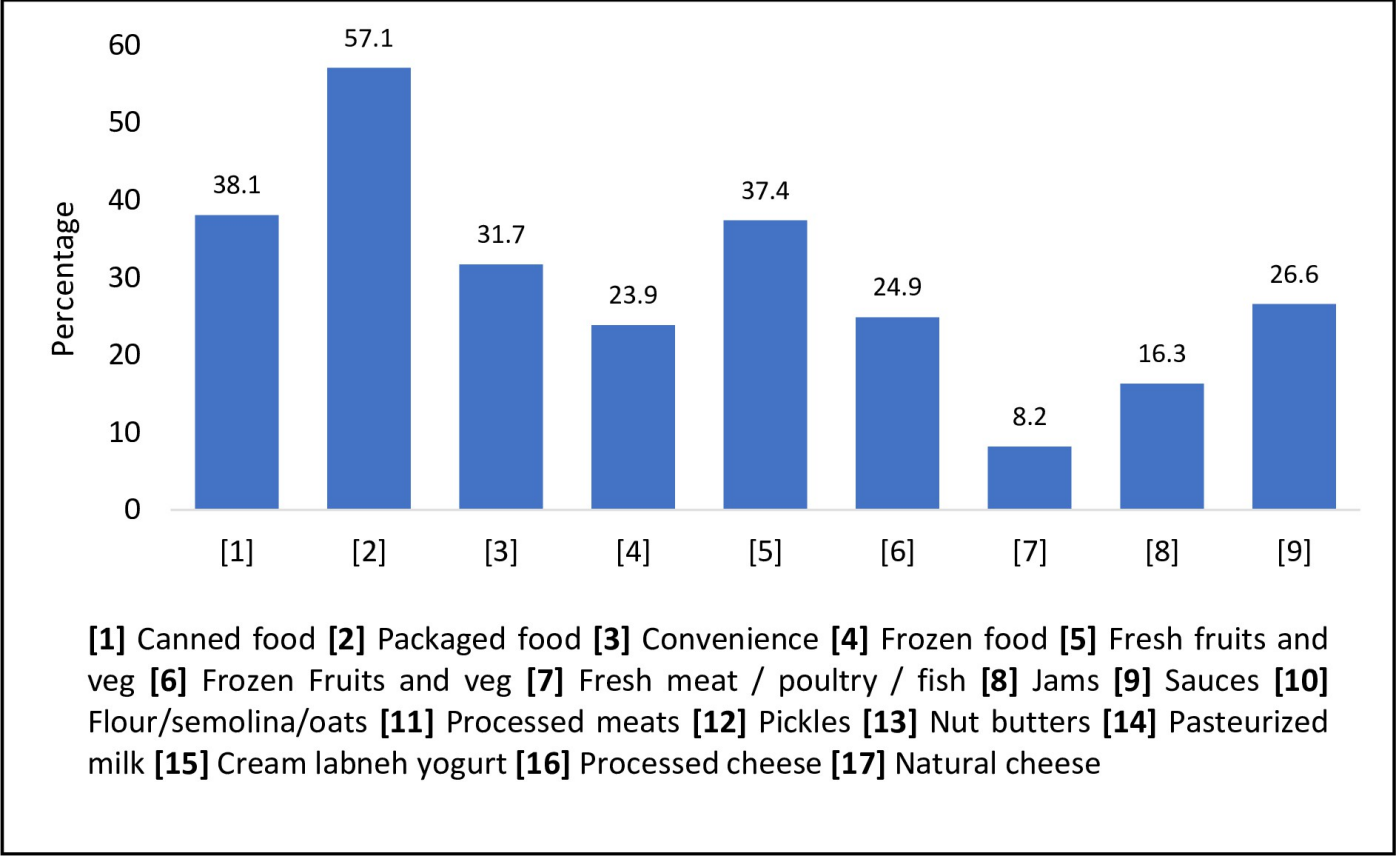
The percentage of respondents who believed about the presence of food addtives in different food items (n = 1038).

In this study, more than half of the consumers did not know that flour/ semolina/ oats (87.4%), pickles (58.3%), nut butters (72.5%), cream/ labneh/ yoghurt (66.8%), and natural cheeses (80.4%) contained FAs (Fig 3). On the other hand, 94.7%, 69.5%, and 94.3% knew that fresh meat/ poultry/ fish, frozen fruits and vegetables, and fresh fruits and vegetables, respectively, did not contain FAs (Fig 3).

About 22% stated that it was not safe to consume FAs despite their levels being within Acceptable Daily Intake (ADI) limits. The FAs of utmost health concern to the respondents were preservatives (68.2%), followed by bleaching agents (51.0%), artificial sweeteners (49.6%), coloring agents (47.7%), flavorings (40.1%), curing agents (35.5%), and antioxidants (22.0%). In a previous study, respondents agreed that FAs were vital for the food industry as they increased the duration of freshness and ease of preparation. However, they also had concerns regarding their ill health effects [25]. Preservatives, artificial sweeteners, coloring, and bleaching agents have been reported to be the most concerning FAs in previous literature [8, 9, 26–29]. It has been reported that even if people were aware that a product contained a FA listed on the packaging, they would still buy it because it is convenient and does not affect flavor [30]. A campaign was conducted on the safety of FAs and it was observed that there was a significant difference in the pre-and post-campaign responses [9]. Prior to this educational campaign, only 14% believed FAs within permissible limits were safe; however, post the campaign, the percentage increased to 74%. This shows the need to educate consumers regarding the safety of FAs to improve consumer trust on the food industry.

### Attitude about FA usage, nature of origin, and disease risk

About 9.7% of the respondents stated that they were not aware of the uses of FAs. The proportion of respondents who reported that the purpose of adding FAs is to extend shelf life, better the taste and aroma of food, enhance nutritional value, improve consistency and texture, and boost appearance and color was 92.1%, 75.0%, 23.5%, 56.6%, and 69.4%, respectively. In a previous study, 93.3% of the respondents reported that FAs are added to increase shelf life, for better taste and aroma (69%), for appearance and color (63.7%) [8]. In contrast, another study reported that only 31.3% of the respondents knew the function of FAs could be preservative in nature [27]. This suggests that variability concerning the knowledge level regarding FAs exists and the general public needs to be educated about the role of FAs to make informed choices. Overall, the ‘naturalness’ of a FA is an important determining factor for acceptability [31].

**Fig 4:**
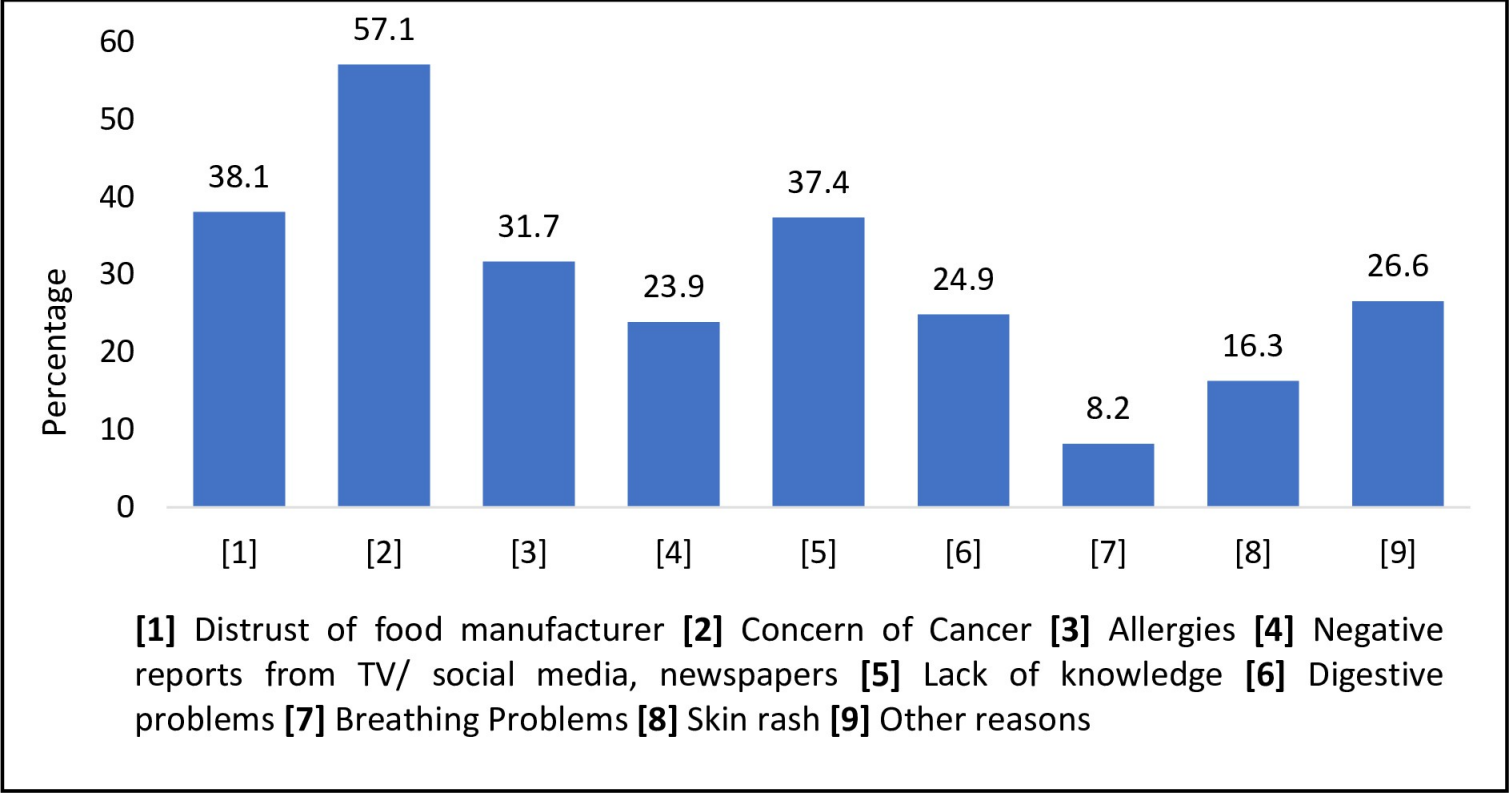
Concerns of the respondents about the safety of food additives (n = 1038).

In the current study, less than half (45.7%) of the respondents were not sure whether legally permitted FAs were safe to consume. Similarly, about 35.0% mistakenly believed that FAs could not have a ‘natural’ origin. A majority (85.3%) thought that food industries used various types of FAs during food production. About half the respondents (52.0%) believed that organic products did not contain FAs and (47.1%) that FAs are not necessarily restricted to packaged ready-to-eat food items only. Only 32.9% of the respondents in this study agreed that food factories complied with government-set food safety standards. A Turkish study reported a staggering 74% of their respondents considered FAs as not safe [8]. In the current study, around 61.4% believed that all FAs were harmful to human health. The respondents believed that the FAs might cause cancer (57.1%), allergies (31.7%), breathing problems (8.2%), and skin rashes (16.3%). Besides this, the lack of trust in the food manufacturer (38.1%), lack of knowledge (37.4%), or negative reports from the media (23.9%) were all considered factors for not trusting the safety of FAs even within safe limits (Fig 4). Negative reports via social media as a reason for lack of trust has been reported previously [9]. A good 76.8% reported that they would check information on food labels in the future. More than half (70.4%) of the consumers said they would appreciate information about FAs if it was provided by the store. This is in line with a previous study conducted in Korea where 73.7% of the respondents reported they would use the information on FAs if provided by the store [9]. This is assuring because it illustrates that consumers are willing to increase their knowledge of FAs and correct their misconceptions.

### Consumer behavior on FA information present on food labels

**Fig 5:**
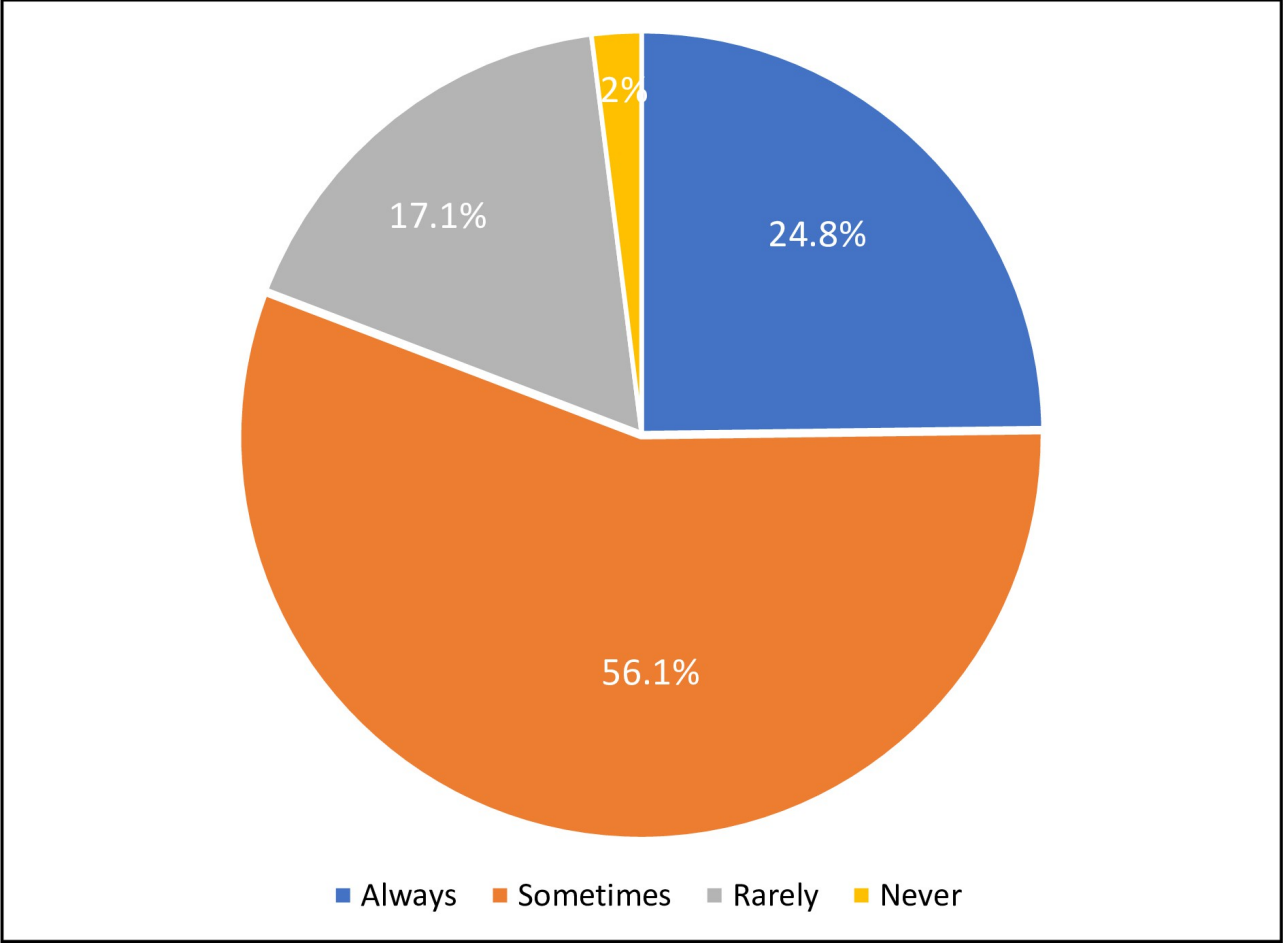
Percentage of respondents who reported reading food labels to check for information on food additives (n = 1038).

About one-fourth of the respondents (24.8%) in this study reported always reading food labels (Fig 5). The frequency of consumption of foods containing FAs indicated that the highest consumption was of table salt (77.3% daily) followed by dairy products (38.2% daily), and the lowest consumption was of processed meats (0.6% daily). It was also observed that most of the respondents did not consume gums like xanthan gum and gum Arabic (55.7%), artificial sweeteners (47.3%), and microwaved popcorn (25.5%). The risk and benefit perception of the consumers is an important factor which determines consumption. It has been observed that consumers are more willing to accept sweeteners as FAs compared to food colorings [13]. Other studies have also reported that the most frequently consumed foods containing FAs were dairy products and snacks [9, 28, 32, 33].

### Consumer preferred information needs regarding FAs

In this study, 60.1% of the respondents reported that food labels did not provide sufficient information about FAs. The insufficiency was determined to be in terms of inadequate knowledge about the FAs (45.7%) followed by difficulty in understanding the subject of FAs (41.6%) and deficient labelling (34.4%). About 8% indicated that they were not interested in the subject. This is in line with a previous study where half of the respondents had an interest in FAs and 71% agreed that they need further information, especially with regard to the safety of FAs [29]. A study conducted in Turkey reported that only 13.3% of their respondents easily understood and read food labels [8]. Meanwhile, a study conducted in Korea observed that 76.8% of the respondents faced difficulties in understanding the subject of FAs [9].

In this study, the most preferred platforms for consumers to receive information about FAs were social media (41.1%), followed by brochures (24.6%), store entrances (16.0%), emails (8.7%), and cashier counters (7.0%). In another study conducted in Korea in 2011, almost half of the respondents chose booklets as their preferred platform to receive information about FAs. Moreover, the study observed that 85.2% of the respondents believed informational materials should be placed next to the display counter [9].

**Fig 6:**
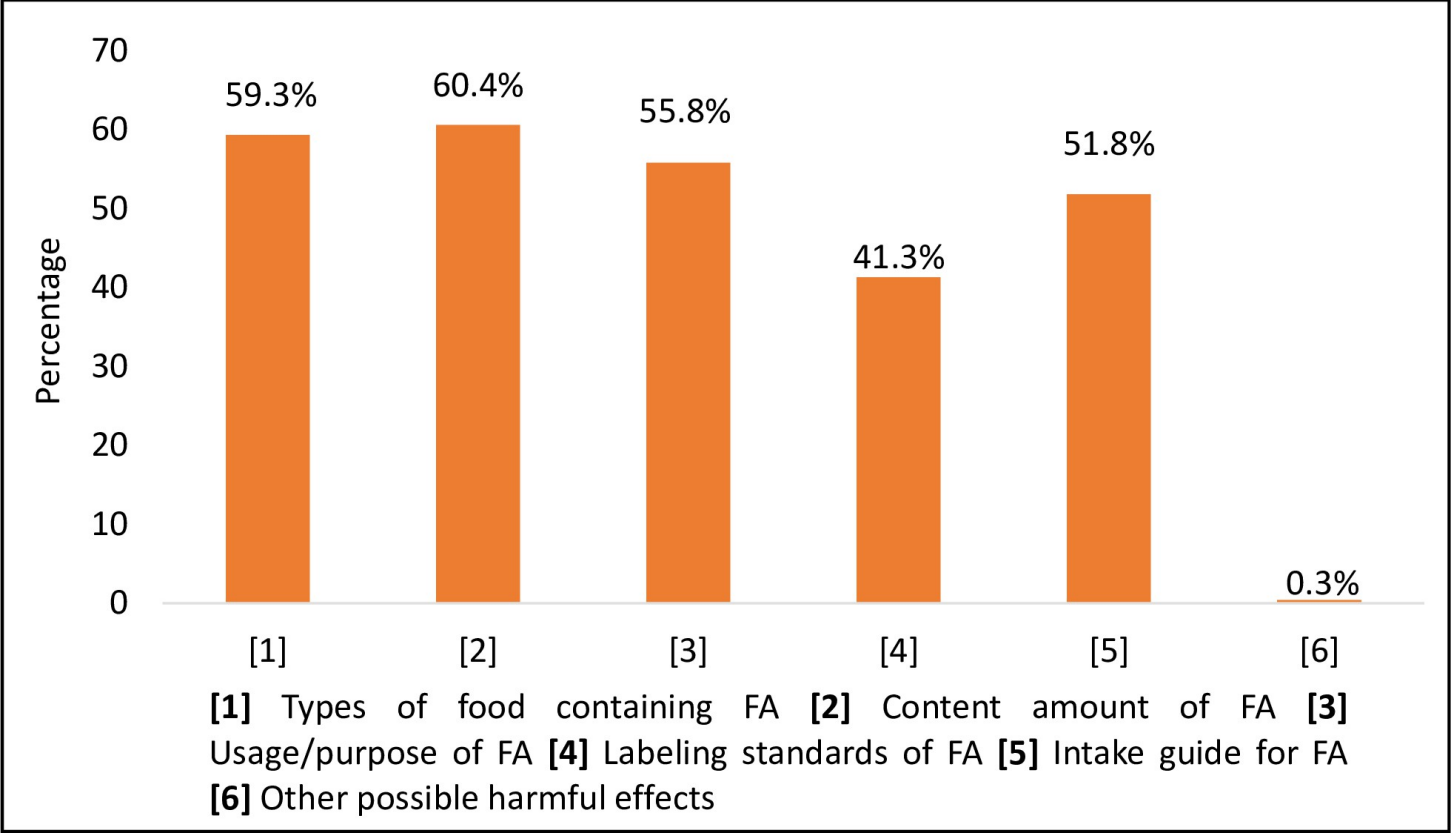
The information about food additives the consumers would like to see (n = 1038).

In terms of the changes that consumers would like food brands to make regarding FAs, 75.3% wanted labels to be designed in a way that they are easier to understand, 41.6% demanded improving legal regulations and standards, 66.2% wanted more information and awareness about FAs while 59.0% wanted the usage of FAs to be reduced. Moreover, more than half the respondents wanted information on the types of foods containing FAs (59.3%), the number of FAs added to the food product (60.4%), usage and purpose of adding the FAs (55.8%), and the intake guide for the FAs (51.8%) to be present on the food label (Fig 6).

### Association of sociodemographic factors with knowledge and attitude score

The average total score in the current study was 29.7 (out of 60) with the minimum being 2 and the maximum being 53. About half of the respondents were classified as having a “good score” based on the criteria mentioned earlier. The score observed in our study is comparatively higher than that observed in Turkey where 37% of the respondents were observed to have sufficient knowledge about FAs [8]. Moreover, in this study females were significantly more likely to have good knowledge and attitude scores as compared to males (*p* = 0.003) (Table 2). Similarly, the age cohort of >60 years was observed to have the greatest scores (*p* = 0.015).

**Table 2:** Association of sociodemographic variables with knowledge and attitude of respondents regarding food additives (n = 1038).

Characteristics		Knowledge & Attitude score		
		Mean	SD	P-value
Gender	Male	28.0	9.7	0.003
	Female	30.1	9.0	
Age	18–24	29.7	9.2	0.015
	25–34	28.2	9.4	
	35–44	30.1	8.6	
	45–60	31.0	9.6	
	>60	33.0	7.5	
Nationality	Arab	29.5	9.1	0.081
	Non-Arab	31.4	9.9	
Education	High school or less	30.8	9.9	0.002
	Bachelor’s	32.2	9.4	
	MSc & PhD	34.6	9.3	
Marital status	Single	29.6	9.0	0.115
	Married	29.5	9.2	
	Widow/ Divorced	33.1	11.0	
Number of children	0	29.3	9.4	0.62
	1	29.0	9.1	
	2	30.8	8.8	
	3	29.9	8.5	
	4	29.6	9.3	
	5 or more	30.1	10.2	

Respondents with a postgraduate degree fared better than their lower educated counterparts (*p* = 0.002). A strong association between the level of knowledge pertaining to FAs and education has been reported previously [8]. In contrast to our findings, a study conducted in China reported that participants with higher education had more concerns about FAs while those with lower education tended to have more faith in the national or international standards [17]. Moreover, the study also reported consumers with low education levels had greater interest and information needs compared to their higher educated counterparts [17]. In another study conducted in Indonesia, it was observed that snack vendors with low levels of education were more likely to sell foods with greater amounts of FAs [34].

The multiple regression analysis indicated that only the level of education (Bachelor’s degree: odds ratio: 1.15 (95% CI: 0.75–1.78); MSc & Ph.D.: odds ratio: 1.74 (95% CI: 1.03–2.93)) had a significant effect on a good knowledge and attitude score (Table 3).

**Table 3:** Multiple regression analysis of the impact of sociodemographic variables on knowledge and attitude of respondents regarding food additives (n = 1038).

Characteristics		Total number (N)	Participants who had good knowledge and attitude		
			n (%)	Odds ratio (95% CI)	P-value
Gender	Male	219	99 (45.2)	Reference	0.145
	Female	819	415 (50.7)	1.26 (0.92–1.71)	
Age	18–24	320	157 (49.1)	Reference	0.343
	25–34	274	122 (44.5)	0.58 (0.17–2.05)	
	35–44	250	129 (51.6)	0.47 (0.13–1.65)	
	45–60	183	99 (54.1)	0.61 (0.17–2.15)	
	>60	11	7 (63.6)	0.65 (0.18–2.33)	
Education	High school or less	95	43 (45.3)	Reference	0.048
	Bachelor’s	777	375 (48.3)	1.15 (0.75–1.78)	
	MSc & PhD	166	96 (57.8)	1.74 (1.03–2.93)	

The results of this study add to the literature of available data regarding knowledge and attitude towards FA. No such study has been conducted previously in the region. It is advisable that university students be knowledgeable about the difference between FA and food adulteration. With all the media hype and wrong information, a well-informed individual with the right attitude would be capable of making correct decisions with respect to the usage of FA or buying of these food products. This would also prevent the general negativity towards products that are processed/exported or imported.

## Limitations

As the study involved recall, it is possible that respondents did not remember the information accurately. Responses may have also been towards the positive for better “social desirability”. Studies designed on observation rather than recall (such as this study) would help overcome this bias. Moreover, only the strata of the society with access to a social media account and the internet could take part in the study. Participants with an internet are expected to have greater access to information about FAs compared to those who do not. Furthermore, the current study design was cross-sectional in nature with no randomization. Participants from each emirate did not have a proportional representation. All these factors affect the generalizability of the findings.

## Conclusion

In our sample, participants who were males, young, and those who had low educational qualifications, were not well educated about FAs. They thereby are expected to be hesitant to purchase a food product with any unfamiliar FAs. There is also a probability that the individuals would also not allow their family members to consume or purchase the food product. Food production company managements or the local municipalities are highly suggested to focus on this stratum to increase the knowledge levels about FAs. Multiple strategies like social media, pamphlets, or brochures can be used as means to disseminate information related to FAs or clear myths surrounding them. This will assist consumers in making informed food choices. Reduced awareness regarding FAs may result in consumers preferring locally produced and fresh foods over imports/frozen or packaged products. This may put undue pressure on the local producers while negatively affecting food manufacturing companies whose food is exported. Further research can be performed to evaluate the efficiency of these interventions in improving food additives knowledge levels. Moreover, a well distributed greater random sample across the Middle East region would further strengthen the findings of the study.

## Supporting information

S1 File Study questionnaire. (DOCX)

## References

[1] FDA. Overview of Food Ingredients, Additives & Colors. 2018 [cited 22 Sep 2021]. Available: https://www.fda.gov/food/food-ingredients-packaging/overview-food-ingredients-additives-colors

[2] Abdulmumeen HA, Risikat AN, Sururah AR. Food: Its preservatives, additives and applications. Int J Chem Biochem Sci. 2012;1: 36–47.

[3] Asghari M, Karimi Zarchi AA, Taheri RA. Preparation and Characterization Nanocrystalline Cellulose as a Food Additive to Produce Healthy Biscuit Cream. Starch‐Stärke. 2021;73: 2000033.

[4] Phillips GO. Acacia gum (Gum Arabic): a nutritional fibre; metabolism and calorific value. Food Addit Contam. 1998;15: 251–264. doi: 10.1080/02652039809374639 9666883

[5] FAO. Codex Alimentarius—International Food Standards. 1995 [cited 24 May 2022]. Available: https://www.fao.org/gsfaonline/docs/CXS_192e.pdf

[6] Parke D V, Lewis DF V. Safety aspects of food preservatives. Food Addit Contam. 1992;9: 561–577. doi: 10.1080/02652039209374110 1298662

[7] Silva M, Lidon F. Food preservatives–an overview on applications and side effects. Emirates J Food Agric. 2016;28: 366–373. doi: 10.9755/ejfa.2016-04-351

[8] Kayışoğlu S, Coşkun F. Determinatıon of the level of knowledge of consumers about food additives. IOSR J Environ Sci Toxicol Food Technol. 2016;10: 53–56.

[9] Shim S-M, Seo SH, Lee Y, Moon G-I, Kim M-S, Park J-H. Consumers’ knowledge and safety perceptions of food additives: Evaluation on the effectiveness of transmitting information on preservatives. Food Control. 2011;22: 1054–1060.

[10] Parlato A, Giacomarra M, Galati A, Crescimanno M. ISO 14470: 2011 and EU legislative background on food irradiation technology: The Italian attitude. Trends food Sci Technol. 2014;38: 60–74.

[11] Migliore G, Thrassou A, Crescimanno M, Schifani G, Galati A. Factors affecting consumer preferences for “natural wine”: An exploratory study in the Italian market. Br Food J. 2020.

[12] Giacomarra M, Crescimanno M, Vrontis D, Pastor LM, Galati A. The ability of fish ecolabels to promote a change in the sustainability awareness. Mar Policy. 2021;123: 104292.

[13] Bearth A, Cousin M-E, Siegrist M. The consumer’s perception of artificial food additives: Influences on acceptance, risk and benefit perceptions. Food Qual Prefer. 2014;38: 14–23.

[14] Varela P, Fiszman SM. Exploring consumers’ knowledge and perceptions of hydrocolloids used as food additives and ingredients. Food Hydrocoll. 2013;30: 477–484.

[15] Ireland J, Rajabzadeh SA. UAE consumer concerns about halal products. J Islam Mark. 2011.

[16] Zhong Y, Wu L, Chen X, Huang Z, Hu W. Effects of food-additive-information on consumers’ willingness to accept food with additives. Int J Environ Res Public Health. 2018;15: 2394. doi: 10.3390/ijerph15112394 30380630 PMC6266858

[17] Wu L, Zhong Y, Shan L, Qin W. Public risk perception of food additives and food scares. The case in Suzhou, China. Appetite. 2013;70: 90–98. 23831014 10.1016/j.appet.2013.06.091

[18] OSU. Snowball Sampling. 10 Apr 2010 [cited 22 Sep 2021]. Available: https://research.oregonstate.edu/irb/policies-and-guidance-investigators/guidance/snowball-sampling

[19] Kadam P, Bhalerao S. Sample size calculation. Int J Ayurveda Res. 2010;1: 55–57. doi: 10.4103/0974-7788.59946 20532100 PMC2876926

[20] Ismail BB, Fuchs R, Mohammad SF. Consumer awareness of the use of additives in processed foods. Ann Food Sci Technol. 2017;18: 316–323.

[21] Borda D, Mihalache OA, Dumitraşcu L, Gafițianu D, Nicolau AI. Romanian consumers’ food safety knowledge, awareness on certified labelled food and trust in information sources. Food Control. 2021;120: 107544.

[22] Coveney J. Food and trust in Australia: building a picture. Public Health Nutr. 2008/03/01. 2008;11: 237–245. doi: 10.1017/S1368980007000250 17672922

[23] Nadia Shaheen Koyratty B, Aumjaud B, Amnee Neeliah S. Food additive control: a survey among selected consumers and manufacturers. Br Food J. 2014;116: 353–372. doi: 10.1108/BFJ-05-2012-0125

[24] Grujić S, Grujić R, Petrović Đ, Gajić J. Knowledge of food quality and additives and its impact on food preference. Acta Sci Pol Technol Aliment. 2013;12: 215–222.

[25] Eiser JR, Coulson NS, Eiser C. Adolescents’ perceptions of the costs and benefits of food additives and their presence in different foods. J Risk Res. 2002;5: 167–176. doi: 10.1080/13669870010004979

[26] Choe J-S, Chun H-K, Hwang D-Y, Nam H-J. Consumer perceptions of food-related hazards and correlates of degree of concerns about food. J Korean Soc food Sci Nutr. 2005;34: 66–74.

[27] Esfahani NB, Ziaei H, Esfandiari Z, Bahreini Esfahani N, Ziaei H. The Knowledge, Attitudes, and Practices toward Food Additives in Personnel of Isfahan University of Medical Sciences in Iran. J Nutr Food Secur. 2021;6: 161–169. doi: 10.18502/jnfs.v6i2.6071

[28] Gökce A, Bozkir C, Seyitoglu DÇ, Pehlivan E, Ozer A. Level of food additive knowledge and perceptions of food safety of university studentsAli Ozer. Eur J Public Health. 2017;27.28355639

[29] Kim H-C, Kim M-R. Consumer attitudes towards food additives. J East Asian Soc Diet Life. 2005;15: 126–135.

[30] Aoki K, Shen J, Saijo T. Consumer reaction to information on food additives: evidence from an eating experiment and a field survey. J Econ Behav Organ. 2010;73: 433–438.

[31] Siegrist M, Sütterlin B. Importance of perceived naturalness for acceptance of food additives and cultured meat. Appetite. 2017;113: 320–326. doi: 10.1016/j.appet.2017.03.019 28315418

[32] Lee CH, Cho YH, Park KH. Assessment of estimated daily intake of nitrite by average consumption of processed foods in Korea. Food Control. 2006;17: 950–956.

[33] Lee J-S. Perception on nutrition labeling of the processed food among elementary school teachers in Busan. Korean J Community Nutr. 2009;14: 430–440.

[34] Dewi NU, Rahman A, Jayadi YI, Hartini DA, Pradana F, Aiman U. Are Sellers Who Have Low level of Knowledge, Attitudes and Practice Selling Snacks With Harmful Food Additives? Indian J Public Heal Res Dev. 2019;10.

