# Supplementary material for: Consumers’ knowledge and attitudes about food additives in the UAE
Source: PLoS One. 2023 Mar 6;18(3):e0282495. doi: 10.1371/journal.pone.0282495 (PMC9987778; doi:10.1371/journal.pone.0282495)
Supplement: S1 File — (DOCX) [file pone.0282495.s001.docx]

# **Supplementary File I**

# **Sociodemographic**

## Age

1. 18-24
2. 25-34
3. 35-44
4. 45-60
5. >60

## Gender:

1. Female
2. Male

## Education:

1. Primary/middle school
2. High school
3. Undergraduate (Bachelor's degree)
4. Postgraduate degree (MSc or PhD)

## Marital status:

1. Single
2. Married
3. Widow
4. Divorced

## Income:

1. I don't have a monthly income
2. < 5,000 AED/month
3. 5,000-10,000 AED/month
4. 10,000-20,000 AED/month
5. 20,000-30,000 AED/month
6. >30,000 AED/month

## Nationality:

1. Arab / Emirati
2. Non-Arab

## No. of children:

1. 0
2. 1
3. 2
4. 3
5. 4
6. 5 or more

# **General**

## What is your source of information about food additives? (Choose all that apply)

### Newspaper or magazines

### TV or broadcasts

### Internet and social media

### Authorities or official information

### Information on food labels

### Food specialists

### Family or friends

### Other

## Rate your level of trust in food labels:

1. Very low trust
2. Low trust
3. Medium trust
4. High trust
5. Very high trust

# **Knowledge**

## Do you know what food additives are?

1. Yes
2. No / Not sure

## Which of the following food additives do you recognize?

- 1. Colorings
  2. Sweeteners
  3. Citric acid
  4. Glucose
  5. Flavorings
  6. Preservatives
  7. Nitrate and nitrite
  8. Emulsifiers
  9. MSG
  10. Antioxidants
  11. Sodium benzoate
  12. Stabilizers
  13. Ascorbic acid
  14. SO2 and sulphites
  15. Viscosity-increasing substances
  16. Sorbic acid
  17. Anti-caking agents
  18. Gelling agents
  19. I don’t recognize any of them

## Which of the following food additives enhances flavour of food?

1. MSG
2. Ascorbic acid/ Beta-carotene/ Not sure

## Which of the following food additives gives gel-like texture to food?

1. Gelatin
2. Dextrin/ Lecithin/ Not sure

## Which of the following is a colouring agent?

1. Nitrates
2. Ascorbic acid/ MSG/ Not sure

## Which of the following foods do you think contains food additives?

### Canned food (corn, mushroom, beans or chickpeas)

### Packaged food (bread, cake mix, biscuits, candies, chocolates, pasta, microwave popcorn, chips, breakfast cereals, instant noodles or instant soups)

### Convenience (ready to eat) food at the grocery store (croissant, cupcakes, cake, sandwiches or a variety of desserts)

### Frozen food (pizzas, samosas, breaded chicken breasts, paneer or casseroles)

### Fresh fruits and vegetables

### Frozen fruits and vegetables

### Fresh meats, poultry or fish

### Jams

### Sauces

### Flour, semolina or oatmeal

### Processed meats (Mortadella, sausage, salami, pastrami or bacon)

### Pickles

### Natural peanut butter, almond butter, or nut butters

### Pasteurized milk or long life milk

### Cream, labneh, fruit yogurt, yogurt or butter

### Processed cheeses (kiri, puk, kraft or la vache qui rit)

### Natural cheeses (cheddar, edam, colby, halloumi, gouda or mozzarella)

## Intake of processed foods containing preservatives is safe if they are consumed within acceptable daily intake.

1. Yes
2. No
3. Not sure

## Which of the following are the most health-concerning food additives?

### Artificial sweeteners

### Preservatives

### Curing agents

### Colorings

### Antioxidants

### Bleaching agents

### Flavorings

### Not sure

# **Attitude**

## Do you think you have good knowledge about uses of food additives?

1. Yes
2. No
3. Not sure

## Do you think that legally permitted food additives are safe?

1. Yes
2. No
3. Not sure

## Do you think most food industries use food additives in foods?

1. Yes
2. No
3. Not sure

## Do you think food additives can be natural?

1. Yes
2. No
3. Not sure

## Do you think organic products contain food additives

1. Yes
2. No
3. Not sure

## Do you think that food additives are only found in packaged ready-to-eat foods

1. Yes
2. No
3. Not sure

## Why do you think food additives are used in food?

### To extend shelf-life

### To give better taste and aroma

### To improve nutritional value

### To give better texture and consistency

### To give better appearance and color

### I'm not sure why additives are added to food

## Do you think food factories conform to government standards of food additives?

1. Yes
2. No
3. Not sure

## Do you think that all food additives are harmful to human health?

1. Yes
2. No
3. Not sure

## If your answer is no or not sure , why do you think legally permitted food additives are not safe

### Distrust of food manufacturers

### Concerns of cancer

### Concerns of allergy

### Negative news report from TV, social media, newspapers

### Lack of knowledge on the subject

### Digestive problems

### Breathing problems

### Skin rashes and swelling

### N/A

## In the future, will you check the information label for food additives when you purchase processed foods?

1. Yes
2. No
3. Not sure

## Would you use extra information about food additives if it is provided to you in the no store?

1. Yes
2. No
3. Not sure

# **Comsumer Behaviour**

## Do you read food labels on food products?

- 1. Always
  2. Sometimes
  3. Rarely
  4. Never

## How often do you consume these products

- 1. Every day
  2. 2-4 times per week
  3. Once a week
  4. Once a month
  5. Rarely
  6. I don't consume them

### Cream, labneh, fruit yogurt, yogurt or butter

### Processed cheeses (kiri, puk, kraft or la vache qui rit)

### Processed meat (salami, pastrami or mortadella)

### Flour, semolina or oatmeal

### Jams

### Sauces

### Canned food (corn, mushroom, beans or chickpeas)

### Frozen meals, heated by microwave/oven or deep-fried (Pizzas, samosas, breaded chicken breasts, sausage, paneer or casseroles)

### Chips

### Ice-cream

### Cookies/crackers

### Chocolates/candies

### Instant noodles/Instant soups

### Stock cubes (Maggi, Knorr)

### Cake mixes

### Pasta

### Microwave popcorn

### Breakfast cereals

### Food colouring agents

### Table salt

### Artificial lemon salt

### Gum Arabic, Guar gum, Carrageenan, Xanthan Gum

### Artificial sweeteners (Sweet 'N Low, Splenda, Nutrasweet, Canderel or Aspartame)

# **Needs**

## Do you find sufficient information about food additives on food labels?

1. Yes
2. No
3. I don’t read food labels

## If your answer is no, why do you think the information about food additives is insufficient? (Choose all that apply) **(1: yes ; 0: no)**

### Insufficient labelling

### Insufficient education

### Difficulties in understanding the subject of food additives

### I am not interested in the subject

### N/A

## To receive information about food additives, which would you choose?

1. Entrance of the store
2. Cashier counter
3. Social media platforms
4. Emails
5. Brochures
6. Other

## What changes would you like food brands to make regarding food additives? **(1: yes ; 0: no)**

### Easy to understand label

### Improved legal regulations and standards of food additives

### More information and awareness on food additives

### Reduce use of food additives

## What Information would you like to see on food labels? **(1: yes ; 0: no)**

### Types of food containing food additives

### Content amount of food additives

### Usage purpose of food additives

### Labelling standards of food additives

### Intake guide for food additives

### Possible harmful effects

## Do you have any safety concerns about food additives?

1. Yes
2. No
